# EFFECTS OF A GAIT TRAINING PROGRAM USING A WALKING-ASSIST WEARABLE ROBOT ON COMMUNITY-DWELLING OLDER ADULTS

**DOI:** 10.1093/geroni/igae098.3548

**Published:** 2024-12-31

**Authors:** Sinwoo Hwang, Eunhee Cho

**Affiliations:** Yonsei University, Seoul, Republic of Korea; Yonsei University, Seoul, Republic of Korea

## Abstract

Two-thirds of people aged 65 and older require help with daily activities such as eating, bathing, and getting in and out of bed or a chair. Walking-assist wearable robots have shown significant improvements in physical function in controlled settings for patients. In this study, we aimed to determine the feasibility and evaluate the effectiveness of a gait assistance and gait resistance training program using a walking-assist wearable robot for community-dwelling older adults. A total of 23 community-dwelling older adults aged 65 and older (30 participants recruited, 7 dropped out) enrolled in a 12-session, 6-week gait assistance and gait resistance training program using a walking-assist wearable robot. A single-group, pre- and post-test design was employed to evaluate effectiveness. Paired t-tests and linear mixed model analysis reveal that the functional performance (assessed using the 10-Meter Walk Test, Timed Up-and-Go, and Four Square Step Test) and leg muscle strength of the participants improved significantly at the end of the program compared to the baseline values (P < 0.01). This study stands out for applying gait assistance and resistance training across various terrains, unlike previous studies that only tested gait assistance in controlled environments. The results demonstrated significant improvements in functional performance and muscle strength in older adults, suggesting the effectiveness of preventive healthcare services utilizing a walking-assist wearable robot as an intervention that can contribute to improving independent functioning and frailty among community-dwelling older adults.

